# The IpaC Carboxyterminal Effector Domain Mediates Src-Dependent Actin Polymerization during *Shigella* Invasion of Epithelial Cells

**DOI:** 10.1371/journal.ppat.1000271

**Published:** 2009-01-23

**Authors:** Joëlle Mounier, Michel R. Popoff, Jost Enninga, Margaret C. Frame, Philippe J. Sansonetti, Guy Tran Van Nhieu

**Affiliations:** 1 Unité de Pathogénie Microbienne Moléculaire, Institut Pasteur, Paris, France; 2 Inserm U786, Institut Pasteur, Paris, France; 3 Unité de Recherche et d'Expertise Bactéries anaérobies et Toxines, Institut Pasteur, Paris, France; 4 Institute of Genetics and Molecular Medicine, University of Edinburgh, Edinburgh Cancer Research Centre, Western General Hospital, Edinburgh, United Kingdom; University of British Columbia, Canada

## Abstract

*Shigella*, the causative agent of bacillary dysentery, invades epithelial cells by locally reorganizing the actin cytoskeleton. *Shigella* invasion requires actin polymerization dependent on the Src tyrosine kinase and a functional bacterial type III secretion (T3S) apparatus. Using dynamic as well as immunofluorescence microscopy, we show that the T3S translocon component IpaC allows the recruitment of the Src kinase required for actin polymerization at bacterial entry sites during the initial stages of *Shigella* entry. Src recruitment occurred at bacterial-cell contact sites independent of actin polymerization at the onset of the invasive process and was still observed in *Shigella* strains mutated for translocated T3S effectors of invasion. A *Shigella* strain with a polar mutation that expressed low levels of the translocator components IpaB and IpaC was fully proficient for Src recruitment and bacterial invasion. In contrast, a *Shigella* strain mutated in the IpaC carboxyterminal effector domain that was proficient for T3S effector translocation did not induce Src recruitment. Consistent with a direct role for IpaC in Src activation, cell incubation with the IpaC last 72 carboxyterminal residues fused to the Iota toxin Ia (IaC) component that translocates into the cell cytosol upon binding to the Ib component led to Src-dependent ruffle formation. Strikingly, IaC also induced actin structures resembling bacterial entry foci that were enriched in activated Src and were inhibited by the Src inhibitor PP2. These results indicate that the IpaC effector domain determines Src-dependent actin polymerization and ruffle formation during bacterial invasion.

## Author Summary

Type III secretion systems (T3SS) are present in a wide range of Gram-negative bacteria that are pathogenic to humans, animals, and plants. These molecular devices allow the injection of bacterial virulence factors into host cells to manipulate various cellular functions. T3SSs share similar functional features. Noticeably, host cell contact triggers the secretion of two T3SS substrates that insert into host cell membranes to form a so-called “translocator” required for the injection of T3SS effectors. *Shigella*, an enteroinvasive pathogen responsible for bacillary dysentery, uses a T3SS to transiently reorganize the actin cytoskeleton and to induce its internalization into epithelial cells. Some *Shigella*-injected T3SS effectors participate in cytoskeletal reorganization, but none of these effectors are totally necessary or sufficient to induce bacterial invasion. We show here that in addition to its role in the injection of bacterial effectors, the translocator component IpaC also induces the recruitment of Src and actin polymerization driving the formation of localized membrane ruffling. Our findings suggest that major signaling through T3S translocator components occurs during the initial steps of bacterial interaction with host cell membranes. Compounds that prevent membrane insertion of the *Shigella* T3S translocator would likely constitute ideal candidates for antimicrobial agents.

## Introduction

*Shigella*, the causative agent of bacillary dysentery, uses a T3S apparatus to invade epithelial cells [1],[2]. Actin reorganization during *Shigella* entry involves Rho GTPases as well as the activation of the Src tyrosine kinase [3],[4]. Using fibroblastic cells derived from Abl knock-out mice, it was shown that Abl-Crk signaling is implicated in the activation of Rac [5], suggesting that Abl activation by Src may act upstream of RhoGTPases. Src tyrosine kinase activity, however, also modulates actin polymerization downstream of RhoGTPases. For example, cortactin, a cytoskeletal protein that has been reported to activate the Arp2/3 complex and to induce actin polymerization, is tyrosyl phosphorylated in a Src-dependent manner during *Shigella* entry into epithelial cells [6]–[8]. In cells where Src activity is inhibited, actin polymerization is limited to the intimate contact between the bacteria and the host cell membrane, indicating that Src allows the amplification of actin polymerization at *Shigella* entry sites required for the formation of extensions that surround the bacterium [3],[8]. Signaling through Src family kinases leading to actin polymerization at the plasma membrane occurs under physiological stimulation or in tumor cells during outside-in integrin receptor-mediated signaling [9]. However, cytoskeletal remodelling dependent on Src has been reported for viruses, bacteria and parasites, suggesting that such signaling corresponds to a common theme that controls actin dynamics at the membrane [9],[10]. Bacterial T3S systems, however, allow the targeting of bacterial pathogenic effectors into the cell cytosol that may potentially bypass membrane signaling [2],[11]. These secretion devices share similar structural and functional features and are widespread among gram-negative pathogens. Upon cell contact, two T3S substrates are secreted and insert into host cell membranes to form a so-called “translocator” that allows the injection of other effectors in the host cell cytosol. Consistently, the *Shigella* IpaB and IpaC translocator components have the ability to form a pore into host cell membranes with an estimated size of 2–3 nm, that corresponds to the estimated inner diameter of the T3S needle [12],[13]. Pathogen-mediated reorganization of the host cell cytoskeleton may occur through translocated effectors that target RhoGTPases, as observed for *Salmonella* or *Yersinia*, or by inducing tyrosine kinase signaling at the membrane in the case EPEC or viruses such as vaccinia [10],[11],[14],[15]. The nucleotide sequence of the *Shigella* genome and of the large virulence plasmid has been determined, providing an exhaustive view of T3S effectors [16],[17]. Among constitutively expressed effectors, some have been implicated in bacterial invasion. The IpaA protein binds to the focal adhesion protein vinculin and induces actin depolymerization, which is required for completion of the entry process [18]. The IpgD protein, that hydrolyses phosphatidylinositol (4,5)-bisphosphate, contributes to the disruption of cortical actin structures required for efficient bacterial uptake [19],[20]. More recently, IpgB1 was reported to act upstream of Rac by hijacking the ELMO/Dock pathway [21],[22]. IpgB2 was identified as a RhoA mimick, but its implication in bacterial invasion has not been investigated [23]. In addition to participating in T3S, IpaC, through its carboxyterminal domain, was shown to induce actin polymerization responsible for the formation of cell extensions that engulf the bacterium [24]–[26]. While *Shigella* type III effectors may act synergistically to induce bacterial invasion, only bacterial mutants for T3S or for translocator components are totally defective for cytoskeletal reorganization and bacterial entry. In this report, we present evidence that the translocator component IpaC, through its carboxyterminus, participates in early signaling events by allowing the recruitment and activation of the Src tyrosine kinase.

## Results

### Src is recruited early during *Shigella* invasion

**Figure 1:**
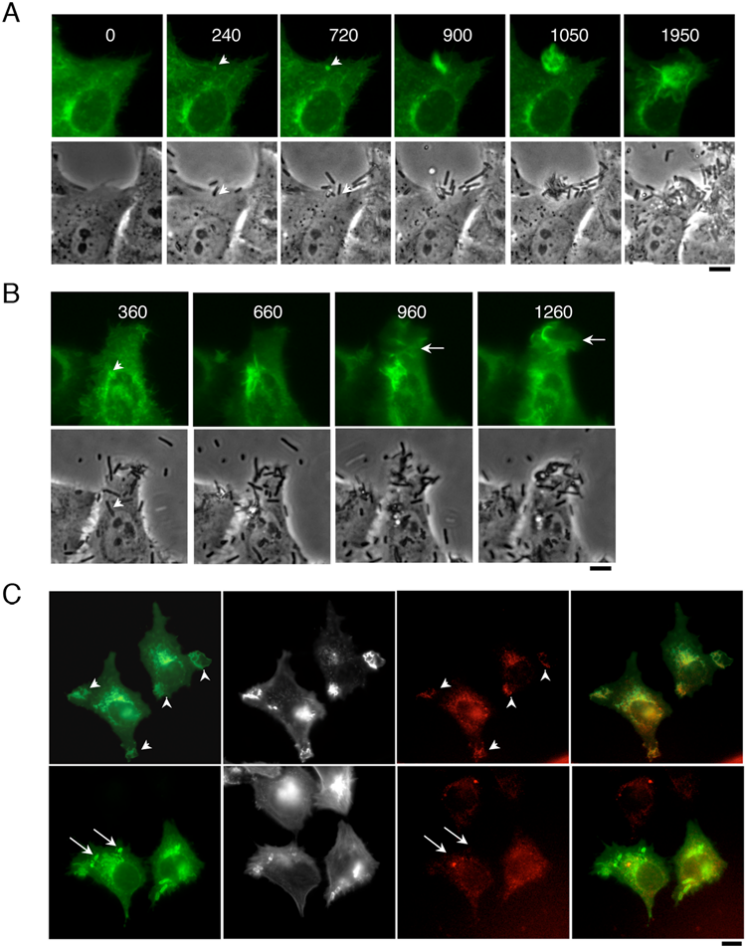
Src is recruited and activated early at *Shigella* entry sites. (A,B) Represent two sequences of bacterial entry events of HeLa cells transiently transfected with Src-GFP. The numbers correspond to the elapsed time from the addition of bacteria in seconds. Src-GFP is recruited early at the intimate bacterial–contact site (arrowheads), followed by recruitment in bacterial-induced ruffles. The arrow points at large ruffles that occur at bacterial entry sites without detectable prior recruitment at the intimate bacterial contact site. Scale bar = 5 µm. (C) HeLa cells transfected with Src-GFP (green) were challenged with bacteria for 15 min at 37°C and processed for immunofluorescent staining of F-actin (greyscale) and anti-PY416 (red). *Shigella*-induced Src activation occurs during bacterial invasion.

To gain insights into the relationship between Src and bacterial induced actin-polymerization, we analyzed the kinetics of Src recruitment during the early phases of bacterial invasion. HeLa cells were transfected with a Src-GFP construct, which was previously shown to behave as endogenous Src [27]. Transfected cells were challenged with *Shigella* while following Src-GFP recruitment and bacterial-induced ruffles by time-lapse microscopy. We selected Src-GFP transfected cells that showed moderate fluorescence levels, with no obvious alterations in their morphology. As shown in Figure 1, Src recruitment often occurred early at the intimate bacterial-cell contact site (Figure 1, arrowheads; see Video S1). Following this initial recruitment, Src was detected in extensions that formed in the vicinity of the adhering bacteria. This pattern of Src recruitment, with an initial recruitment at the intimate bacterial contact site, was observed in the majority of foci analyzed (68%, 22 foci, n = 3). In other instances, however, Src-containing ruffles formed in a more diffuse manner, without obvious correlation with an invading bacterium and appeared to merge over the cell surface to form large ruffles (Figure 1B, arrows). Anti-pY416, an antibody that recognizes the active form of Src family kinases (Materials and Methods) showed staining at *Shigella* actin foci, suggesting Src activation at entry sites (Figure 1C, arrowheads). Following bacterial invasion, however, Src was found recruited at the intimate bacterial contact site inside host cells without detectable pY416 staining (Figure 1C, arrows), suggesting that Src activation occurs transiently at the levels of bacterial-induced entry foci. Consistently, *Shigella* invasion induced a transient Src-dependent tyrosyl phosphorylation of the cytoskeletal protein cortactin, correlating with actin foci formation and peaking at 15 min that was strongly inhibited by the Src inhibitor PP2 (Figure S1).

To determine whether other kinases could participate in *Shigella*-induced actin polymerization, which could account for the formation of entry foci with no detectable recruitment of Src, we used SYF fibroblastic cells that are deficient for all three Src family kinases, Src, Fyn and Yes [27]. When challenged with the *Shigella* wild-type strain, SYF cells formed small actin foci that were not detected when cells were challenged with the isogenic non-invasive *mxiD* strain (Figure S2A and S2B), suggesting that other kinases that do not belong to the Src family kinases could participate in *Shigella* invasion. Transfection of Src-GFP in these cells, however, resulted in an increase in the size of *Shigella*-induced actin foci, as well as in a three-fold increase in the frequency of actin foci formation (Figure S2A and S2B). In contrast, Fyn-GFP transfection did not result in any significant increase in foci formation (Figure S2A and S2B). Furthermore, while Src-GFP recruitment could be observed at the bacterial contact site in 73.0%±6.6 (SEM) of the cells analyzed (100 cells, n = 3), only 3.6%±1.3 (SEM) showed Fyn-GFP recruitment at the bacterial contact site (584 cells, n = 3) (Figure S2C). Together, these data support a major role for Src in *Shigella*-induced actin polymerization during invasion, and suggest that PP2 – sensitive kinases other than Src family kinases could also participate in *Shigella* invasion.

### Early recruitment of Src at *Shigella* entry sites occurs independently of actin polymerization

**Figure 2:**
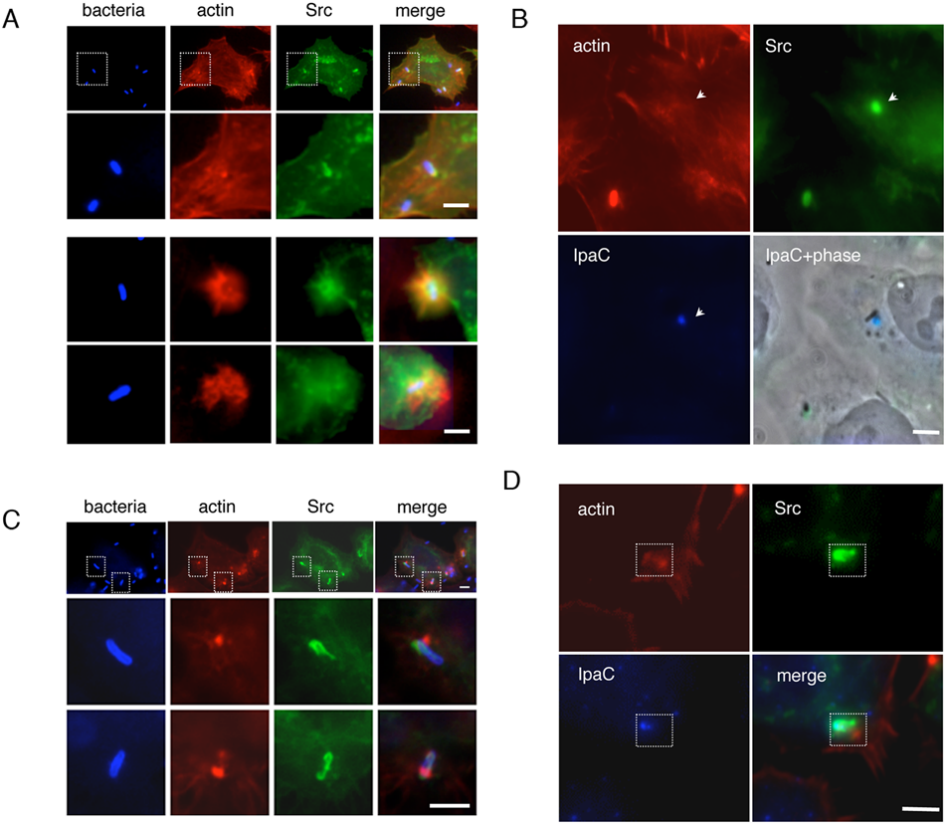
Differential pattern of Src and actin localization at *Shigella* entry sites. HeLa cells transfected with Src-GFP were challenged at 37°C with the wild-type strain M90T. (A,B) Reconstructions of deconvolved images from focal planes spaced by 0.2 µm. Samples were fixed and processed for fluorescent staining with rhodamine-phalloidin (actin), anti-bacterial LPS (blue). GFP-fluorescence is shown in green. (A) Cells were challenged with bacteria for 15 min. Src localizes in an inner region of foci, whereas F-actin labels bacterial-induced ruffles (arrowheads). (B) Cells were treated with cytochalasin D at a final concentration of 1 µg/ml for 30 min and challenged with bacteria for 10 min in the absence of cytochalasin D (Materials and Methods). Src-GFP is recruited at the bacterial-cell intimate contact, whereas *Shigella*-induced actin polymerization is detected as individual patches juxtaposing the bacteria (arrowheads). (C,D) HeLa cells transfected with Src-GFP were challenged for 10 min at 37°C with the *ipaC*/C1 strain. Samples were fixed and processed for fluorescent staining with rhodamine-phalloidin (red), and anti-IpaC pAb (blue). GFP-fluorescence is shown in green. (C) Cells treated with cytochalasin D at a final concentration of 1 µg/ml for 30 min prior to bacterial challenge. Src, actin, and IpaC: deconvolved images of a single focal plane; IpaC+phase: overlay of the IpaC panel with the corresponding focal plane acquired in phase contrast (Materials and Methods). During early stages of bacterial invasion, IpaC is detected at bacterial-cell contact sites and colocalizes with Src-GFP, in a region where little F-actin is detected. Scale bar = 5 µm.

To analyze the localization of Src relative to polymerized actin, dual fluorescent labeling was performed. In nascent entry sites, Src was detected at the close bacterial-cell contacts (Figure 2A, top panels). In more developed entry foci, while polymerized actin was detected in bacterial-induced extensions, Src localized in an inner region containing little F-actin in 67.2±11.4% of foci (45 foci, n = 3), as illustrated in Figure 2A, arrowheads. In the remaining foci, Src localized in extensions but not in strict association with actin as shown in Figure 2A, bottom panels, suggesting that Src recruitment could occur independently of actin polymerization. To test this, cells were treated with the actin polymerization inhibitor cytochalasin D prior to bacterial challenge. Control experiments indicated that cell treatment with cytochalasin D for 30 min at a final concentration of 1 µg/ml led to the disappearance of actin stress fibers, indicating efficient actin inhibition (not shown). Such treatment followed by bacterial challenge in the presence of cytochalasin D led to virtually total inhibition of *Shigella*-induced actin polymerization ([28]; not shown). Removal of cytochalasin D after cell treatment by successive washes prior to bacterial challenge (Materials and Methods) however, allowed the detection of de novo polymerized actin, which was visualized as distinct patches at the bacterial vicinity (Figure 2B). These patches could clearly be distinguished from Src recruitment at the intimate bacterial contact site (Figure 2B). Very few bacterial-induced actin rich ruffles could be observed and Src recruitment remained limited to the intimate bacterial-cell contact sites (Figure 2B), suggesting that the distribution of Src away from the attached bacteria required actin polymerization.

To analyze the role of T3S in Src recruitment, we performed immunofluorescent staining of the translocator component IpaC. As shown in Figure 2C, IpaC staining localized with Src in areas where little F-actin could be detected after 5 min of *Shigella* challenge at 37°C (Figure 2C, arrowheads). At these early stages of invasion, however, bacterial-induced actin polymerization is not prominent, making it difficult to assess whether T3S is linked with foci formation. When samples were incubated for 15 min at 37°C, bacterial-induced actin foci were clearly observed, but IpaC was only detected in approximately 20% of these entry foci analyzed suggesting a highly dynamic distribution of IpaC during foci formation. In all foci analyzed, IpaC staining was detected in areas of the foci where Src was enriched (39 foci, n = 4), corresponding to an inner region of the actin foci (Figure S3). When cells were treated with cytochalasin D, IpaC could still be detected at the bacterial contact site where Src was enriched (Figure 2D, dashed box).

Together, these results suggest that the early distribution of Src at the bacterial intimate contact occurs independent of actin polymerization, however the distribution of Src away from the bacterial attachment site requires actin polymerization as this was not observed in the presence of cytochalasin D.

### T3S ‘early invasion’ injected effectors are dispensable for Src recruitment and activation during *Shigella* invasion

**Figure 3:**
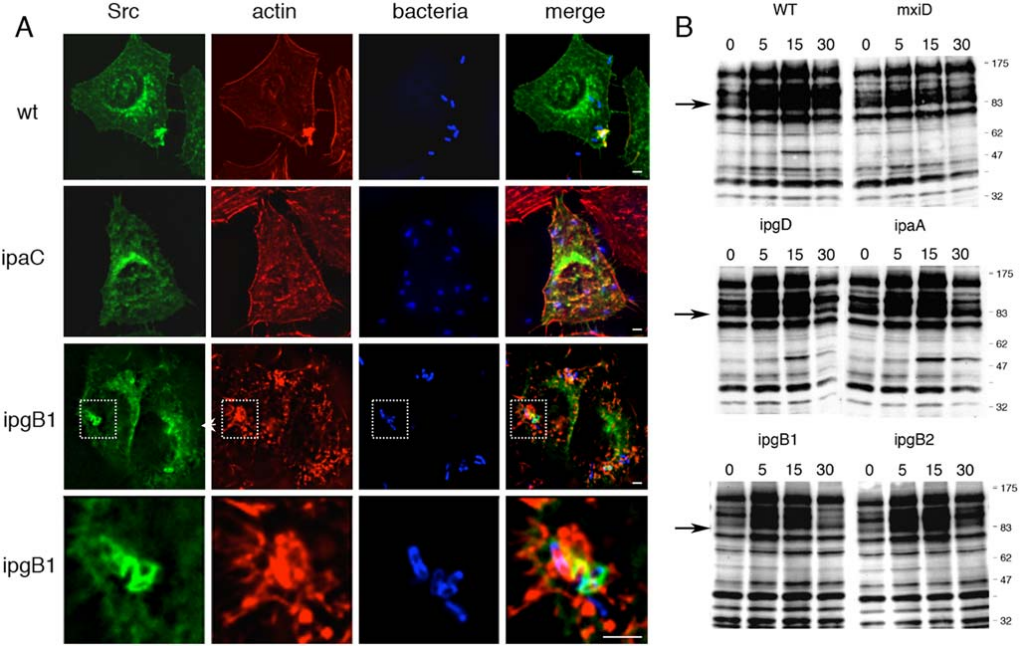
*Shigella* T3S translocated IpgB1 effector is not required for Src recruitment and activation. (A) HeLa cells were transfected with Src-GFP and challenged at 37°C for 15 min. with wild-type *Shigella*, the *ipaC*, or the *ipgB1* mutant strains. Samples were fixed and processed for anti-LPS immunofluorescence labelling (blue), and F-actin staining (red). GFP fluorescence is shown in green. The panel corresponds to reconstructions of deconvolved images from focal planes spaced by 0.2 µm. Unlike the *ipaC* mutant, the wild-type and *ipgB1* mutant induces Src recruitment at bacterial entry sites. (B) HeLa cells were challenged with the indicated *Shigella* strains, and at various time intervals samples were lysed and processed for anti-phosphotyrosine Western-blot analysis (Materials and Methods). The time is indicated above each lane in min. The *Shigella* mutant strains deficient for the translocated T3S effectors *ipaA*, *ipgD*, *ipgB1*, and *ipgB2* induce phosphotyrosine patterns that are similar to that induced by wild-type, whereas the *ipaC* mutant is defective at inducing tyrosine phosphorylation. The arrows indicate the expected migration of tyrosylphosphorylated cortactin.

To investigate the relationship between translocated T3S effectors and *Shigella*-induced Src-signaling, we analyzed the recruitment of Src in cells challenged with various *Shigella* strains. As shown in Figure 3A, wild-type *Shigella* or an isogenic *ipgB1* mutant strain induced Src recruitment at entry sites (Figure 3A, wt and ipgB1). In the case of *ipgB1*, however, Src recruitment remained at the close vicinity or the intimate bacterial-cell contact and was not observed in membrane ruffles surrounding the bound bacteria as wild-type *Shigella*. We extended this analysis to all other *Shigella* translocated T3S effectors previously been implicated in bacterial invasion. *Shigella* mutants for IpaA, IpgB2, and IpgD were fully proficient at recruiting Src at bacterial entry sites (Figure S4). In contrast, the translocator component mutants of IpaB or IpaC failed to induce Src at bacterial contact sites (Figure 3A, ipaC, Figure S4). As previously described, when anti-phosphotyrosine Western-blot analysis was performed on cells challenged with wild-type *Shigella*, a clear induction of protein tyrosylphosphorylation was observed that was not detected when cells were challenged with the non-invasive isogenic derivative mxiD mutant strain (Figure 3B) [3]. As shown in Figure 3B, the various *Shigella ipaA*, *ipgD*, *ipgB1* or *ipgB2* mutant strains induced patterns of tyrosine phosphorylation, in particular the Src-dependent phosphorylation of the 80 kDa cortactin protein, that were not different than those induced by wild-type *Shigella* (Figure 3B, arrows). Collectively, these results indicate that T3S translocated effectors of invasion are dispensable for Src recruitment and activation during *Shigella* invasion.

### Effects of IpaC mutations on bacterial-induced hemolysis and invasion

**Figure 4:**
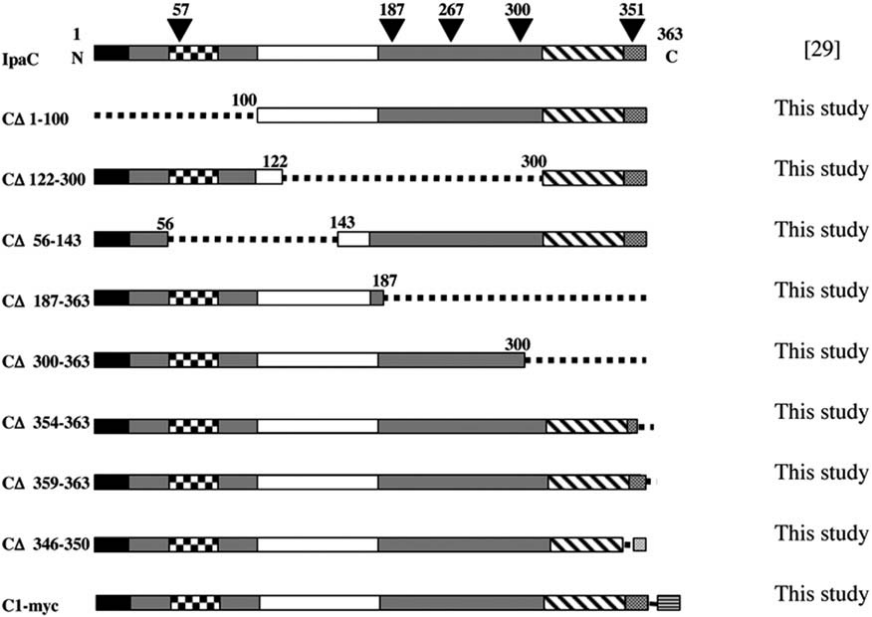
Scheme of IpaC derivatives. Domain organization of IpaC. Solid black box: secretion signal sequence; checked box: chaperone binding region; empty box: hydrophobic predicted transmembrane domain; hatched box: coiled-coil region; dotted-box: region involved in actin polymerization and bacterial invasion. The name of IpaC derivatives is indicated on the left. The solid triangles represent the insertion of an 11-residue epitope from phage C3 within the *ipaC* coding sequence [29]; the position of the residue corresponding to the insertion site is indicated. Deletions corresponding to the indicated residues are represented by a dotted line. The myc epitope insertion at the carboxyterminal end is represented by a grey box with horizontal lines.

As bacterial mutants for T3S translocated effectors still induced Src signaling, we then investigated whether the translocator components could be involved in this process. In particular, because IpaC had been previously involved in signaling leading to actin polymerization, we analyzed the effects of IpaC on Src activation induced by *Shigella* during entry. Biochemical and structure-function studies of IpaC have provided insights about its domain organization [26],[29],[30]. As depicted in Figure 4, IpaC contains a large hydrophobic domain (residues 100–170) allowing interaction with lipid membranes, and a carboxyterminal coiled-coil domain (residues 330–357) that is involved in oligomerization and in actin nucleation [26]. To differentiate between mutations in IpaC that inhibit signaling but not T3S translocation, we constructed a series of deletions and analyzed them for their ability to complement an *ipaC* mutant for red blood cells hemolysis, an assay previously used to test for T3S-induced pore formation (Figure 4; [12]). As shown in Table 1, and as expected, virtually no hemolysis was observed for the T3S-defective *mxiD* mutant, while the *ipaC* mutant showed decreased hemolytic activity of 16.5% efficiency. Complementation of the *ipaC* mutant with full length IpaC restored hemolytic activity to levels comparable to those induced by wild-type *Shigella*. In contrast, the *ipaC* internal deletions CΔ122–300, CΔ56–143, CΔ300–363, CΔ187–363 or removal of the last five or ten carboxy-terminal residues in CΔ359–363 and Δ354–363, respectively, strongly reduced bacterial-induced hemolysis (Table 1). Deletion in CΔ346–350 had little effects on hemolysis or actin foci formation. Interestingly, while in general, deletions had drastic effects on hemolysis, insertion of an eleven-residue epitope at position 300 (C300) only partly reduced hemolysis, while insertions at positions 57 (C57) or 351 (C351) of a C3 epitope, or of a Myc-epitope C-terminal to IpaC, still show significant bacterial-induced hemolytic activity (Table 1). In spite of these high levels of hemolytic activity, C300 and C351 showed a strong defect in bacterial invasion, with levels that were not different than those observed in an *ipaC* mutant (Table 1). Together, these data support the notion that the last 63 carboxyterminal residues are involved in signaling leading to actin remodeling and bacterial invasion.

**Table 1:** Effects of *ipaC* mutations on *Shigella*-induced contact hemolysis.

Strain	Secretion^a^	Relative % Hemolysis^b^	Actin Foci/Cell^c^	% Bacterial Invasion^d^
M90T	+	96.0±3.2	1.2	100
mxiD	−	5.0±0.3	0.0	0.09±0.02
ipaC	−	16.5±5.7	0.0	0.21±0.08
ipaC/pC1	+	93.0±6.7	0.4	47.7±1.56
ipaC/pCΔ1–100	−	ND	ND	ND
ipaC/pCΔ122–300	+	3.3±0.7	0	ND
ipaC/pCΔ56–143	+	4.3±0.5	0	ND
ipaC/pCΔ300–363	+	12.1±4.1	0	ND
ipaC/pCΔ187–363	+	8.2±1.4	0	ND
ipaC/pC57	+	95.5±5.0	1.1	34±9.80
ipaC/pC187	+	95	0.3	20.55±0.15
ipaC/pC267	+	95	0.4	26.25±3.88
ipaC/pC300	+	49.2±3.7	0.1	0.25±0.05
ipaC/pC351	+	88.7±2.2	0.0	0.25±0.04
ipaC/pC1myc	+	70.7±5.7	0	ND
ipaC/pCΔ346–350	+	90	1.2	ND
ipaC/pCΔ359–363	+	12	0	ND
ipaC/pCΔ354–363	+	12	0	ND

a Bacterial strains were grown to mid-exponential phase in the presence of Congo Red, and *ipaC* transformants were induced with IPTG to allow the expression and the secretion of the various IpaC constructs (Materials and Methods). Secretion was determined by analyzing the presence of the IpaC derivative in the bacterial culture supernatant by anti-IpaC Western-blot analysis. − : no detectable IpaC in the bacterial culture supernatant; + : detection of the IpaC derivative. No significant variation in the efficiency of secretion could be observed between the various constructs (not shown), with the exception of the CΔ1–100 construct that does not show significant levels of secretion.

b Horse blood erythrocytes were challenged with the various bacterial strains, and the extent of hemolysis was quantitated by reading the absorbance at 595 nm (Materials and Methods). The relative percentage of hemolysis corresponds to the ratio of the hemolysis determined for the sample over that of total hemolysis, determined for samples treated with 0.1% SDS. The values correspond to the mean of three independent experiments performed in triplicates. The *ipaC* mutant strain shows residual hemolytic activity that was previously shown to depend on IpaB [12]. The *ipaC* transformants expressing the C57, C351, or C1myc derivatives show a hemolytic activity that is comparable to that conferred by full-length IpaC.

c HeLa cells were challenged with bacterial strains for 15 min. at 37°C. Samples were fixed and processed for fluorescent staining of F-actin and bacterial LPS. Bacterial induced actin foci were scored microscopically, and the numbers were normalized to the number of cells analyzed. The values are representative of at least 40 cells analyzed in a representative experiment.

d Bacterial internalization was measured using the gentamicin assay. The values correspond to representative experiments performed in triplicates.

### *Shigella* invasion does not require high levels of T3S substrates translocation

**Figure 5:**
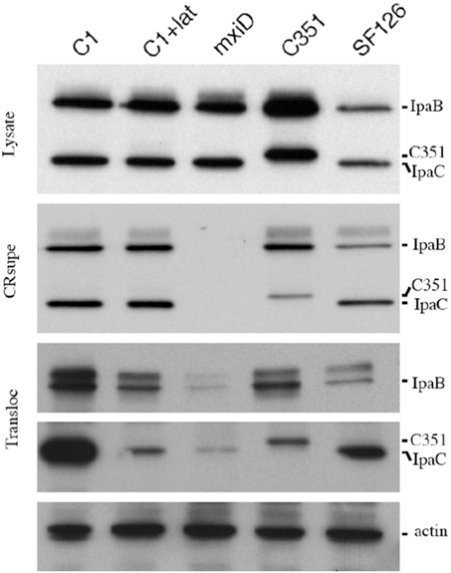
The C351 insertion in the IpaC effector domain does not prevent T3S translocation. Bacterial strains were grown to exponential phase, and T3S was induced by the addition of Congo Red in the culture medium. Bacterial lysates (Lysate) or supernatants (CRsupe) were analyzed by anti-IpaB and anti-IpaC Western blot analysis. HeLa cells were challenged with the various bacterial strains for 30 min at 37°C. Translocated T3S effectors were subjected to immuno-precipitation and analyzed by Western-blot analysis (Transloc, Materials and Methods) or by anti-actin Western-blot analysis (actin). The corresponding T3S effectors are indicated on the right. Cells challenged with: C1: *ipaC*/pC1; C1+lat: *ipaC*/pC1 after latrunculin treatment; mxiD: the non-invasive *mxiD* strain; C351: *ipaC*/pC351; SF126: a polar insertion upstream of the ipa locus that leads to decreased expression of Ipa proteins [31].

**Figure 6:**
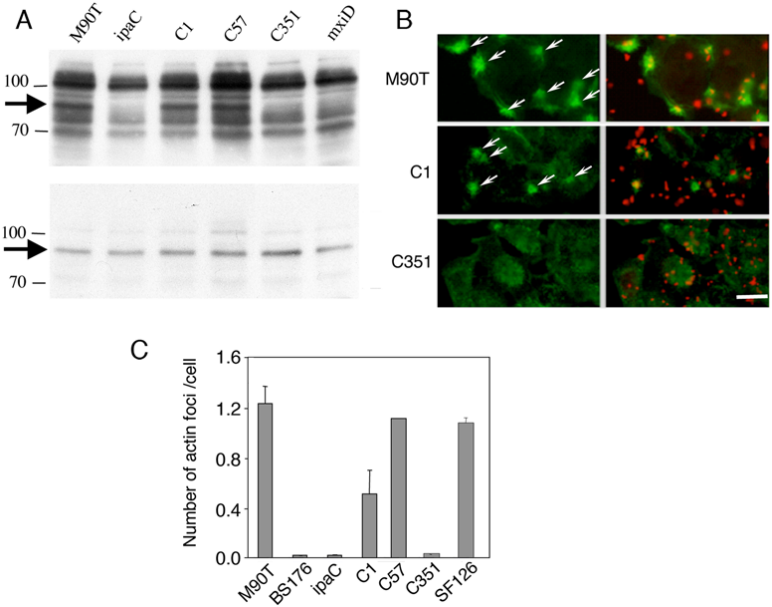
The C351 insertion in the IpaC effector domain impairs Src-dependent signaling. (A) HeLa cells were challenged for 15 min at 37°C with the bacterial strain indicated above each lane. Cell lysates were subjected to anti-phosphotyrosine (top panel) or anti-cortactin (bottom panel) Western blot analysis. The molecular weight markers are indicated. The arrows point at cortactin. As oppposed to C57, C351 do not complement the *ipaC* mutant strain for induction of the phosphorylation of cortactin. (B) HeLa cells were challenged for 15 min at 37°C with bacteria. Samples were fixed and processed for fluorescent staining with rhodamine-phalloidin (green) and anti-bacterial LPS (red). Arrows indicate actin foci. Scale bar = 10 µm. (C), Actin foci were scored microscopically on at least 4 independent experiments (n>200). Cells challenged with the indicated strains, or with the *ipaC* mutant complemented with C1, C57, or C351. SF126 harbours a polar insertion upstream of the *ipa* locus that leads to decreased expression of Ipa proteins [31]. Expression of the C57 construct allows the *ipaC* mutant to induce actin foci at the same frequency as the wild-type strain, whereas expression of full length IpaC leads to actin foci that are smaller and less numerous than those observed with wild-type *Shigella*. Very few actin foci could be observed for the *ipaC* mutant strain expressing the C351 insertion.

To investigate what were the limiting levels of translocated T3S effectors for bacterial invasion, we performed translocation assays in HeLa cells. Experiments were conducted with strain SF126, a *Shigella* mutant strain that expresses reduced levels of Ipa proteins but that still efficiently invades epithelial cells [31]. Briefly, following bacterial challenge, samples were lysed in 0.1% Triton, under conditions that do not induce bacterial lysis. After elimination of intact bacteria by centrifugation, cell lysates were subjected to anti-IpaB, or -IpaC immuno-precipitation and immunoprecipitates were analyzed by Western-blotting. As shown in Figure 5, in the *ipaC* mutant complemented with wild-type IpaC, secretion of IpaB and IpaC in host cell membranes could be readily detected (Figure 5, C1). As expected, no translocation could be detected for the T3S-deficient *mxiD* mutant (Figure 5, mxiD). As shown in Figure 5 and in line with their respective levels of expression, the SF126 strain showed levels of translocation of IpaB and IpgD that were strongly reduced compared to *ipaC*/pC1 (Figure 5, Transloc). In spite of these low levels of T3S translocation, however, and consistent with previous reports, SF126 was as efficient as wild-type *Shigella* in inducing actin foci formation (Figure 6) [31]. Furthermore, actin polymerization and Src recruitment at entry sites induced by SF126 were not different than those induced by wild-type *Shigella* (Figure S4). These results indicate that only a minor fraction of translocated T3S substrates is required for bacterial invasion.

### The C351 insertion impairs signaling, leading to actin reorganization and bacterial invasion

We then investigated whether impairment of bacterial invasion for the *ipaC*/pC351 was linked to a defect in the translocation of T3S substrate. As shown in Figure 5, although *ipaC*/pC351 showed levels of translocation that were reduced compared to those occurring in the *ipaC*/pC1 strain expressing wild-type IpaC (Figure 5, Transloc, compare C351 and C1), translocated IpaB and IpaC351 were clearly detected when compared to the T3S-deficient *mxiD* strain (Figure 5, Transloc). IpaB and IpgD were translocated at levels that were even higher in the *ipaC*/pC351 strain than in the invasive proficient strain SF126 (Figure 5, Transloc). Consistent with its inability to induce signaling required for actin reorganization, anti-phosphotyrosine Western-blot analysis performed on lysates of cells infected with the various *ipaC* complemented strains indicated that *ipaC*/C351 was as deficient as the *ipaC* or *mxiD* mutants at inducing Src-dependent cortactin phosphorylation (Figure 6A) and at inducing actin foci (Figure 6B and 6C). In contrast, while *ipaC*/pC57 was as efficient as wild-type, *ipaC*/pC351 was as deficient as the *ipaC* or *mxiD* mutants to induce actin foci formation. These results indicate that the C351 insertion does not affect the translocation of T3S effectors to levels that are detrimental for *Shigella* invasion. Similar results to C351 were obtained with the C1myc construct (data not shown). Both *ipaC*/pC351 and *ipaC*/pC1myc strains were deficient at inducing actin foci and at recruiting Src (Table 1 and Figure S5).

Together, these results support a direct role for the carboxyterminal domain of IpaC in signaling leading to bacterial uptake.

### The actin polymerization inhibitor Latrunculin B inhibits the translocation of *Shigella* type III effectors

**Figure 7:**
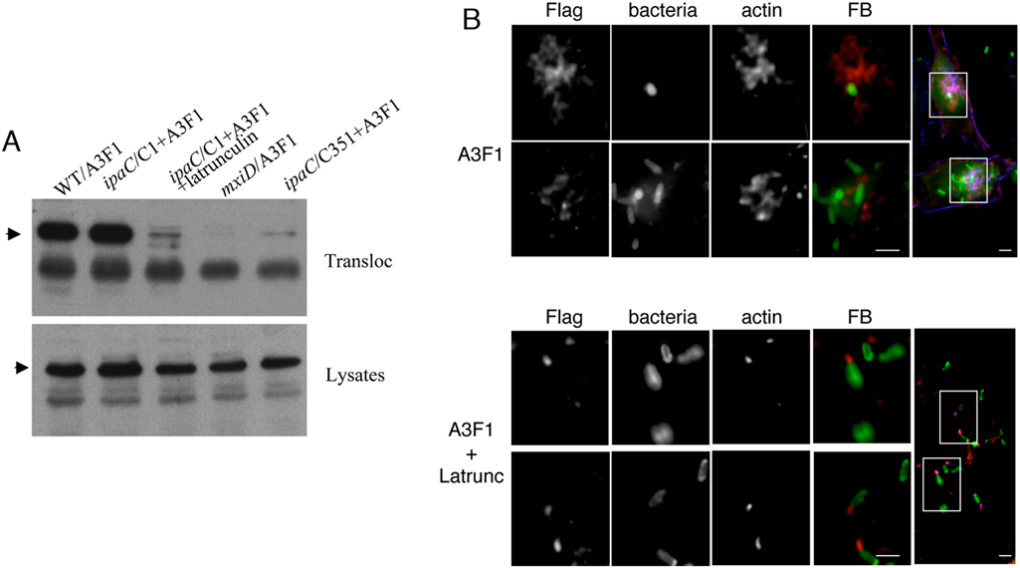
Cell treatment with the actin polymerization inhibitor Latrunculin B leads to decreased T3S translocation. (A) HeLa cells were challenged with A3F1-expressing bacteria for 30 min at 37°C, and translocated A3F1 was subjected to anti-IpaA immunoprecipitation and analysed by anti-FLAG Western-blot analysis (Transloc). In control experiments, total lysates corresponding to bacteria-infected cells were analyzed by anti-FLAG Western blot (Lysates). A3F1: bacteria expressing the A3F1 construct. WT: wild type *Shigella*; *ipaC*/C1: ipaC mutant complemented with full length IpaC; mxiD: a T3S defective isogenic mutant; *ipaC*/C351: *ipaC* mutant expressing C351. The arrows point at the A3F1 construct. (B) HeLa cells were challenged with wild-type *Shigella* expressing A3F1 for 15 min at 37°C in the absence (A3F1) or presence of latrunculin A (A3F1+Latrunc). Samples were fixed and processed for immunofluorescence staining of bacterial LPS (bacteria), FLAG epitope (Flag), and actin (actin). FB: bacteria are in green and the FLAG staining is in red. The panels represent reconstructions of deconvolved images from focal planes spaced by 0.2 µm. The insets in the right panels are shown. Scale bar = 5 µm. Translocation of A3F1 is reduced upon latrunculin treatment.

Because the C351 insertion that impaired *Shigella*-induced actin polymerization led to reduced translocation, we investigated whether actin polymerization was required for efficient T3S translocation. Cells were treated with latrunculin to inhibit bacterial induced actin polymerization and the levels of translocated effectors were determined. As shown in Figure 5, latrunculin treatment significantly reduced translocation of IpaB and IpaC in the *Shigella ipaC*/pC1 strain, down to levels that were comparable to those observed in *ipaC*/pC351. In control experiments, latrunculin treatment had no effects on IpaB and IpaC expression levels or their in vitro secretion (Figure 5). To determine the effects of latrunculin on the translocation of injected effectors, we constructed a T3S reporter IpaA derivative, in which the carboxyterminal IpaA-vinculin binding region was replaced by a triple-FLAG epitope (A3F1) that we expressed in wild-type *Shigella*, the *ipaC*/pC1, or the *ipaC*/pC351 strains (Materials and Methods). As shown in Figure 7A, translocated A3F1 could clearly be detected in cells challenged wild-type *Shigella*/pA3F1 or in *ipaC*/pC1 pA3F1. In contrast, A3F1 showed decreased levels of translocation in *ipaC*/pC351 pA3F1 (Figure 7A). As observed for IpaB and IpaC, latrunculin treatment decreased A3F1 translocation for *ipaC*/pC1 pA3F1 to similar levels as observed for *ipaC*/C351. These findings were confirmed by anti-FLAG immunofluorescence staining of A3F1. When cells were challenged with wild-type *Shigella*/A3F1, A3F1 staining could be visualized throughout actin foci. Latrunculin treatment, however, inhibited actin polymerization and limited A3F1 to discrete spots contacting the bacteria (Figure 7B).

These results indicate that actin polymerization during bacterial invasion is required for efficient T3S translocation. Taken together with data from the previous section, these results suggest that the C351 insertion in the IpaC carboxyterminal effector domain impairs the translocation of T3S effectors because it interferes with IpaC-mediated actin polymerization.

### The IpaC carboxyterminal effector domain induces Src-dependent actin polymerization

**Figure 8:**
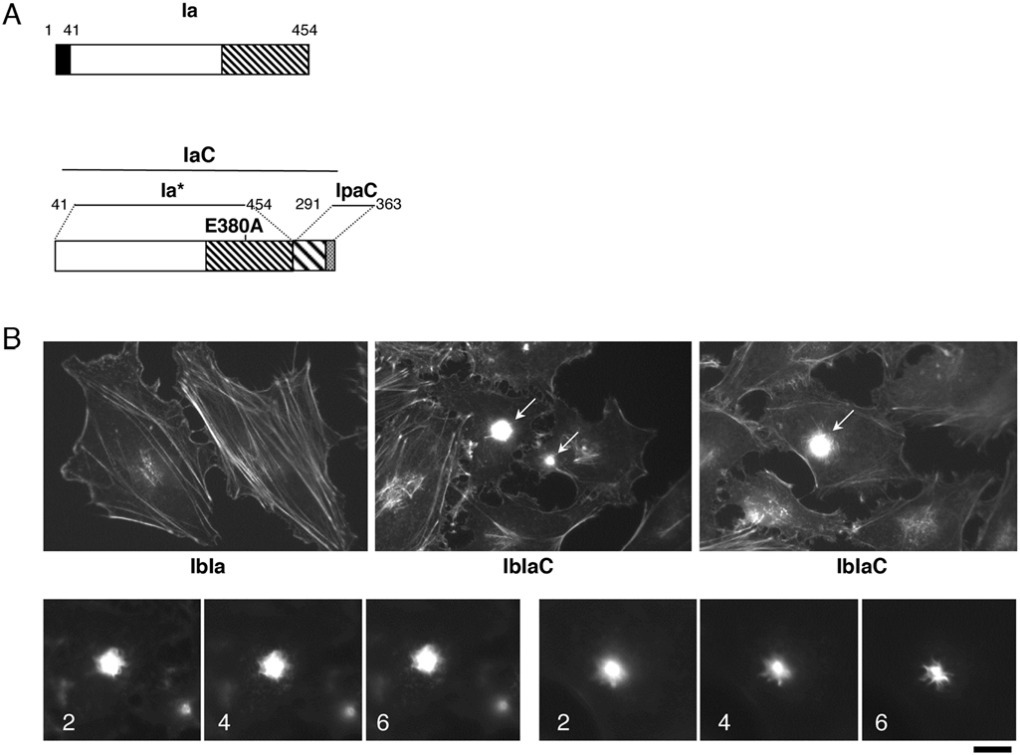
The IpaC last 72 carboxyterminal residues fused to the Iota Ia component induces actin foci-like structures. (A), Schematic construction of IaC. Ia, the Iota Ia component. Solid box: signal sequence. Hatched box: enzymatic domain. IaC, the carboxyterminal residues 291–363 of IpaC containing the effector domain were fused to Ia*, corresponding to residues 41–454 of Ia with the E380A substitution that inactivates its enzymatic function [32]. (B) HeLa cells were challenged with the Iota Ib component and Ia* (IbIa) or IaC (IbIaC and bottom panels) and processed for fluorescent staining of F-actin. Arrows point at IbIaC-induced actin ruffles. Bottom panels: two series of focal planes corresponding to IbaC-induced actin foci. The numbers indicate the distance in µm from the cell basal surface.

**Figure 9:**
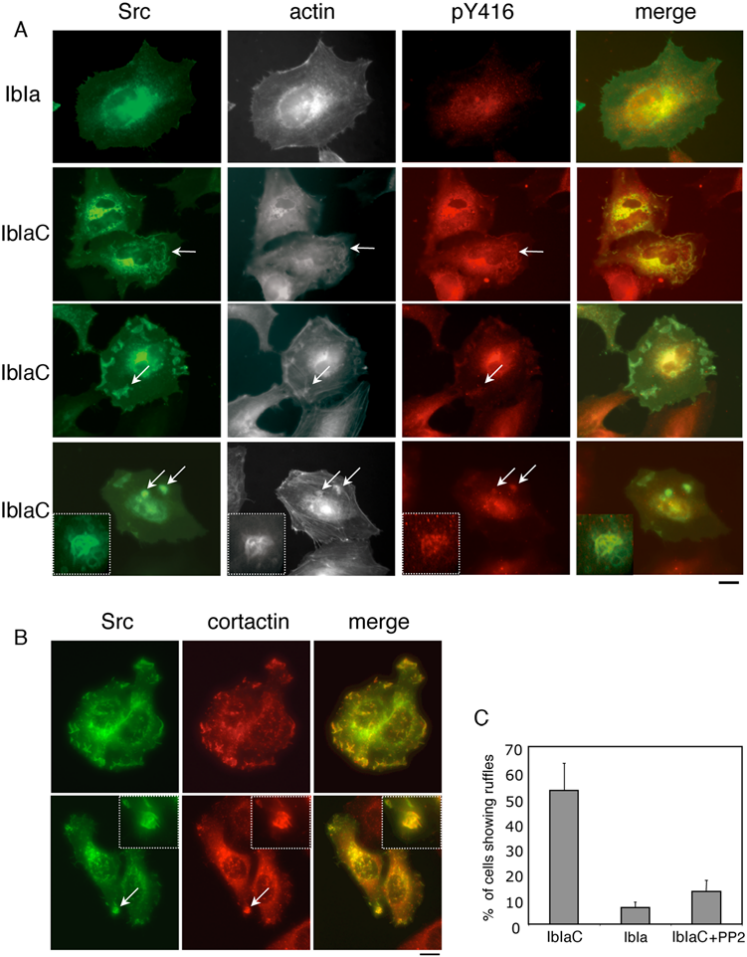
IaC-induced actin structures are enriched in Src. HeLa cells transfected with Src-GFP were challenged with the Iota Ib component and Ia* (IbIa) or Ic (IbIaC) and processed for immunofluorescence staining of F-actin and PY416 (A) or cortactin (B). Arrows point at IbIaC-induced actin ruffles that contain activated Src. Actin foci detected in IbIc-stimulated cells are enriched in Src-GFP and cortactin (arrows). Scale bar, 5 µm. (C) Cells were challenged with IbIaC, IbIa, or IbIaC in the presence of 10 µM PP2. Src-GFP transfectants showing ruffles were scored microscopically. The numbers are representative of at least 300 transfected cells in three independent experiments.

We next wanted to determine whether the IpaC effector domain alone was sufficient to induce Src activation and actin polymerization. In previous studies, using a cell permeabilization assay, IpaC was shown to induce actin polymerization and filopodial structures [24]. This assay, however, did not allow us to study Src activation as the permeabilization procedure by itself induced changes in the pattern of tyrosyl phosphorylation (Mounier, unpublished data). We therefore sought a system that does not involve cell permeabilization and that, unlike cell transfection, would allow us to study short-term effects on cytoskeletal reorganization mediated by the translocation of the IpaC effector domain at the cell membrane. To do this, we used the Iota two-component toxin as a delivery system [32]. The IpaC carboxyterminal residues 291–363 containing the oligomerization and effector domain were coupled to the residues 41–454 of the Iota Ia component. A glutamic acid was substituted for an alanine residue at Iota Ia position 380 to abolish its ADP-ribosyl transferase activity to generate the IaC peptide (Figure 8A; [32]). This construct was expressed and purified as a His-tagged fusion recombinant protein (Materials and Methods). Cells were incubated with the Ib and IaC components for 15 min at 21°C, followed by 15 min at 37°C (Materials and Methods). Samples were fixed and processed for fluorescence staining of F-actin. As shown in Figure 8B, control cells treated with IbIa showed a defined cortical actin periphery with prominent stress fibers (Figure 8B, IbIa). In contrast, incubation with IbIaC led to disappearance of stress fibers and the formation of actin-rich microspikes at the cell periphery (Figure 8B, IbIaC). Strikingly, in a few instances, actin-rich structures that resembled bacterial-induced entry foci could also be observed on the apical surface of IbIaC treated cells (Figure 8B, arrows and bottom panels). Like bacterial entry foci, these structures were raised several microns above the cell surface and consisted of actin-rich membrane leaflets that appear to emanate from a discrete area (Figure 8B). When quantified, 17. 0±1.0% of IbIaC-treated cells showed these actin foci. In contrast, no such actin structures were detected when cells were treated with Ib and Ia alone (300 cells, n = 3), or with Ib and IaC351, which corresponds to recombinant Ia fused to the IpaC carboxyterminal residues 291–363 containing the C351 insertion (395 cells, n = 3) (Materials and Methods, Figure S6). To detect the IbIaC complex, Ib was fluorescently labeled using as previously described [33]. Fluorescent Ib showed a staining reminiscent of intracellular endosomes consistent with previous studies using this delivery system (not shown and [33]). However, when cells were incubated with fluorescent Ib associated with IaC, staining could also be detected at the levels of actin-like foci enriched in Src-GFP (Figure S7, arrows). To determine the role of Src in actin structures induced by IaC, cells transfected with Src-GFP were treated with IbIaC and processed for immunofluorescence staining of F-actin and with anti-pY416. As shown in Figure 8A, in transfected cells, IbIaC induced the formation of large ruffles enriched in Src-GFP and F-actin (Figure 9A, arrows). In addition to these ruffles, IbIaC also induced actin foci that contained Src-GFP (Figure 9B). These IbIaC-induced structures were also enriched in activated Src as indicated by staining with the anti-pY416 antibody (Figure 9A) and cortactin (Figure 9B). In contrast, and as expected, Src-GFP transfected cells challenged with IbIa showed fewer ruffles, with 6.6±2.1%, compared to 49.6±15.9% of IbIaC treated cells with ruffles (Figure 9A and 9C). When treated with PP2, a significant inhibition of ruffles formation was observed following IbIaC stimulation, with only 13±4.3% of the cells displaying ruffles (Figure 9C). Taken together, these results indicate that the effector domain of IpaC is sufficient to induce Src-dependent actin ruffles.

## Discussion

We show here that Src is recruited at the intimate contact site between *Shigella* and the host cell membrane during the early steps of invasion. Shortly after this intimate recruitment, however, Src was observed in extensions surrounding the bacteria, and cells pre-treated with cytochalasin had no Src accumulation in these extensions. This suggests that Src activation occurs at the intimate bacterial-contact site and that Src rapidly diffuses from this site in an actin-dependent manner. This is reminiscent of a report in which Src activation was followed in real time using a fluorescence resonance energy transfer probe and anti-integrin coated beads as a means to trigger local Src activation [34]. It was observed that following Src activation at the site of bead-membrane contact, Src activation rapidly diffused from the initial site of activation in an actin-dependent manner and filopodial extensions formed at the cell periphery at sites distal from the bead [34]. In previous reports, we showed that Src inhibition did not prevent actin polymerization at the intimate bacterial-cell contact, but that the Src-dependent cortactin phosphorylation was required to amplify actin polymerization at extensions surrounding the invading microorganism [3],[8]. Thus, actin polymerization that leads to bacterial-induced ruffling appears to require Src activation, but Src also needs actin polymerization to localize properly in ruffles that surround the bacteria. Such a dual requirement for Src and actin has been reported in processes controlling cell adhesion dynamics and Src activation [27],[35].

Consistent with a role for IpaC in Src recruitment and activation, IpaC often co-localized with Src at entry sites, although in many instances, Src could be detected at entry sites in the absence of IpaC. This could reflect the rapid dynamics of Src diffusion, as discussed above, or alternatively, could correspond to removal of IpaC from entry structures linked to its degradation, as shown for the *Salmonella* T3S effector SopE [36]. The IpaC effector domain localised to the last 63 C-terminal aminoacids of the protein, was also reported to direct actin nucleation *in vitro* [26]. In this study, IpaC rarely co-localized with F-actin, contradicting the notion that it acts simply as an actin nucleator. Also, the fact that the *ipaC*/C1 strain that secretes larger amounts of IpaC but induces actin foci of smaller size than wild-type *Shigella* argues against a simple direct role of IpaC in actin nucleation. Rather, these results indicate that a proper balance of IpaC-mediated signaling determines optimal actin polymerization.

We show that *Shigella* T3S is dramatically enhanced by actin polymerization, given that latrunculin treatment significantly reduced the translocation of T3S effectors. A role for actin in T3S has also been observed as *Yersinia* T3S-dependent pore formation is sensitive to actin inhibitors [37]. Since the Rho but not the Rac GTPase was found to be required for the translocation of Yop proteins, it was speculated that actin polymerization mediated by formins downstream of Rho was important for T3S-mediated injection of effectors [38]. One possibility is that following the initial bacterial-cell contact and a primary injection of T3S effectors, actin polymerization allows the formation of cell extensions that favors further contact between the plasma cell membranes and T3S apparatus, thus increasing T3S. Consistent with this hypothesis, real time analysis of *Shigella* invasion indicates that the initial contact between bacteria and the host cell implicates only a limited area at the bacterial surface, often at one bacterial pole, while bacterial engulfment involves the formation of membrane ruffles along the bacterial sides ([39]; Video S2).

To follow effects linked to translocation of the IpaC carboxyterminal effector domain into the cell cytosol, we used the *C. perfringens* Iota two-component toxin, which together with the related anthrax toxin has been previously used as intracellular delivery systems [32],[40],[41]. Iota belongs to the ADP-ribosylating family of toxins, sharing the same transport mechanism as the anthrax toxin [42]. Following association with the binding component and cellular internalization, the enzymic component is presumed to translocate into the cell cytosol through the pore formed by the heptameric binding component [42]. The small 2–3 nm diameter pore suggests that the enzymic component needs to unfold to translocate [42],[43]. The *Shigella* T3SS needle is thought to have an internal diameter of 2–3 nm, and it is also believed that IpaC needs to unfold to transit through the needle. Thus, it is possible that this type of delivery system involves few structural constraints and could be used with other T3S effectors. Consistent with translocation occurring early after endocysosis at the levels of early endosomes [44], this system allowed the visualization of Src-dependent ruffles induced by the IaC fusion within minutes following addition to cells. Strikingly, cell delivery of IaC also led to the occasional formation of discrete actin structures resembling bacterial entry foci, showing recruitment of active Src. These structures are virtually never found in unstimulated HeLa cells, suggesting that the use of the Iota delivery system to translocate the IpaC effector domain presents similarities with T3S-mediated translocation. Among these similarities, cell stimulation by IaC presumably occurs at the levels of host membranes in a synchronous manner. In the case of invading bacteria, however, cytoskeletal reorganization is linked to the rapid and localized injection of T3S effectors [39],[45],[46]. It is possible that these are limiting factors in the case of Iota-mediated IaC translocation that restrict the frequency of actin foci formation. Also, *Shigella*-mediated ruffles may implicate other T3S effectors such as IpgB1 that favors actin foci formation around invading bacteria [21],[22]. Why does IaC induce the formation of actin foci, even though its localization is not restricted to these foci? The reason for this is unclear, however one possibility is that some sub-cellular areas are more proficient at inducing actin foci because they are enriched in a particular component associated with the plasma membrane, required for localized ruffles. Thus IaC endocytosed at these “proficient” areas would trigger the formation of actin foci, while endocytosis in other areas would be non-productive. When performing live imaging of *Shigella* T3S during cell challenge, we observed that bacterial-induced actin foci formation implicated at least one bacteria triggering T3S at the corresponding site [46]. Consistent with cell surface heterogeneity in its ability to form actin foci, bacterial injection of T3S effectors was not systematically accompanied by actin reorganization (our unpublished results).

Phosphorylation provides a means to insure the regulation of transient events by regulating intra- and inter-molecular association. [47]–[49]. Src phosphorylation also controls the scaffolding of macromolecular complexes involved in cell adhesion or motility whose function may depend on the intensity or the nature of the stimuli. Deciphering the precise mechanism involved in IpaC-mediated activation of Src will provide insights into mechanisms controlling actin dynamics at the membrane.

## Materials and Methods

### Antibodies and reagents

The 4G10 anti-phosphotyrosine monoclonal antibody was from Upstate Biotechnology Inc, (Lake Placid, NY). Horse blood was obtained from Biomérieux (Marcy l'Etoile, France).

Rabbit polyclonal sera against *S. flexneri* LPS or IpaC were described previously [50]. The anti-Src monoclonal antibody clone 327 was purchased from Oncogene Science (Uniondale, NY), and the anti-Src polyclonal antibody N16 was from Santa Cruz Biotechnology. Anti-rabbit antibody coupled to CY3 was from Jackson ImmunoResearch and anti-mouse coupled to Alexa Fluor 488 or −380 were from Molecular Probes. The anti-FLAG M2 mAb, Phalloïdin coupled FITC was from Sigma corp. (St-Louis, MO).

### Bacterial strains, growth conditions, and cell lines

M90T was used as a wild-type invasive strain of *Shigella flexneri* serotype 5. *Shigella mxiD* is a non-invasive mutant of M90T in which the *mxiD* gene has been inactivated and which is deficient for cell contact-dependent secretion. An *ipaC* mutant strain was used for complementation by plasmids producing IpaC-C3 recombinant proteins. The recombinant proteins were constructed by inserting the C3 epitope-coding sequence in the gene *ipaC* carried by plasmid pC1 [29]. Strain SF126 has an insertion in *icsB*, that shows a polar effect on the downstream *ipa* genes and lead to a reduction in Ipa proteins expression without detectable effect on bacterial invasion [31]. Bacterial strains were grown in trypticase soy broth at 37°C with agitation. For invasion assay, overnight cultures were diluted 100-fold and grown at exponential phase (0D_600 nm_ = 0.3). Bacteria were washed in PBS and resuspended in DMEM (Dulbecco Modified Earl's Medium 1 g/l glucose) (Gibco BRL) containing 50 mM HEPES (N-(2-Hydroxyethyl)piperazine-N′-(2-ethanesulfonic acid) pH 7.4 (Sigma corp.). HeLa cells (ATCC # CCL, 2), an epithelial cell line from human cervical carcinoma, were grown at 37°C, 10% CO_2_ in DMEM supplemented with 10% fetal calf serum.

### Plasmid constructions

The pC1 plasmid containing the cloned *ipaC* gene, as well as the C3-containing insertions were described previously [51]. The plasmid containing internal truncations of *ipaC*, constructs pCΔ56–143 and CΔ122–300, were generated from pC1 after digestion with *BclI* and *PstI*, or *PstI* and *NsiI*, respectively, end-filling and religating. The amino-terminal deletion CΔ1-100 was generated by amplifying *ipaC* using the 5′-forward primer 5′-GAGC CAT ATG CCA GAG AAC ACT CTG GAT-3′ and the reverse primer 5′-GCGA GGA TCC TTA AGC TCG AAT GTT ACC-3′: the PCR fragment was digested with *NdeI* and *BamHI* and cloned in the corresponding sites of pET19b (invitrogen). The carboxy-terminal deletions CΔ187–363 and CΔ300–363 were generated by digesting pC1 with *NsiI* or *AflII*, respectively, end filling and religating. The C1-myc construct was obtained amplifying and cloning the *ipaC* ORF including an upstream *Shine Dalgarno* sequence into the *NheI* and *XbaI* sites of the pBAD18 vector using the primers 5′-ATATGCTAGCAAGGAGATATACATATGGAAATTCAAAACACAAAACC- 3′ and 5′-CGCTCTAGAAGCTCGAATGTTACCAGCAAT-3′. Subsequently, a phosphorylated oligonucleotide duplex encoding the myc sequence was inserted in frame into the *XbaI* and *HindIII* sites (5′primer: CTAGAGAACAGAAACTGATTAGCGAAGAAGATCTGTAAA; 3′primer: AGCTTTTACAGATCTTCTTCGCTAATCAGTTTCTGTTCT). The A3F1 construct was obtained by digesting plasmid pBad∶IpaA [52] by *PvuII* and *KpnI* to remove the last 3′-end 85 nucleotides of *ipaA* that encodes the major IpaA vinculin binding site [53],[54]. The following primers 5′ –TGGATTATAAAGATGACGATGACAAGGATTATAAAGATGACGATGACAAGGATTATAAAGATGACGATGACAAGTAACTGCATAAGGTAC – 3′ and 5′-CTTATGCAGTTACTTGTCATCGTCATCTTTATAATCCTTGTCATCGTCATCTTTATAATCCTTGTCATCGTCATCTTTATAATCCA-3′ were annealed, filled in, digested with *PvuII* and *KpnI* and the DNA fragment was cloned into the corresponding sites of pBad18∶IpaA [51] to generate the A3F1 construct fused to a triple-FLAG epitope. A3F1 was excised from pBad∶IpaA using *EcoRI* and *KpnI* and subcloned into the corresponding sites of pSU18 that is compatible with pUC-based plasmids to generate pSU∶A3F1. To construct the IaC fusion, the *SalI-XhoI* fragment of plasmid pMRP516 containing the DNA insert encoding the C3 exoenzyme was exchanged for a 216 bp PCR product encoding the IpaC residues [291–363] using the 5′-TTTTGTCGACTATCCACATCAGGAGGG-3′ and 5′-GCGACTCGAGTTAAGCTCGAATGTTAC-3′ primers. The IaC351 fusion was constructed using the same strategy and primers but using the pC351plasmid as a matrix for PCR amplification. All clones were verified by DNA sequencing.

### Bacterial invasion and anti-phosphotyrosine immunoblot

Bacteria were grown at OD_600_ = 1, and freshly coated with poly-L-lysine before challenge to facilitate bacterial adhesion to cells. Bacteria were washed with PBS and resuspended with poly-L-lysine (Sigma corp.) at a final concentration of 10 µg/ml. After 10 min incubation at room temperature, bacteria were washed 3 times with PBS, resuspended in DMEM containing 50 mM HEPES pH 7.4, and used immediately. HeLa cells were plated the day before at semi-confluency (5×10^5^ cells/35-mm diameter culture dish), were washed twice with PBS containing 1 mM sodium orthovanadate and challenged with the bacterial suspension for 20 minutes at room temperature to allow bacterial attachment, and incubated for 15 min at 37°C. Samples were transferred on ice, washed twice in ice-cold PBS containing sodium orthovanadate and scraped with a rubber-policeman in 100 µl of Laemmli loading sample buffer. Samples were loaded onto SDS-PAGE gels, and analyzed by anti-phosphotyrosine Western blot. Filters were processed with ECL detection kit (Amersham corp.). When indicated, cells were treated with 10 µM PP2 for 30 min at 37°C in DMEM containing 50 mM HEPES pH 7.4, and challenged with bacteria in the same buffer.

### Contact hemolysis

Contact hemolysis analysis was performed as described previously [12] with some modifications. Horse blood erythrocytes were washed in PBS and resuspended at 7.10^8^ cells/ml. Bacteria were grown to late exponential phase, washed in PBS and resuspended at 10^10^ bacteria/ml at room temperature. 50 µl of the bacterial suspension were mixed with 50 µl of erythrocytes in a round bottom 96-well plate. Samples were centrifuged at 150×g for 15 minutes (Rotixa/RP, Hettich, Germany) and incubated at 37°C for 90 min. Samples were resuspended in 150 µl of PBS and centrifuged for another 15 min. 100 µl of the supernatant were transferred to a new plate. The extent of erythrocytes lysis was determined by reading the optical density at 595 nm [12].

### Translocation of T3S effectors assay

Analysis of translocated T3S effectors was performed as described previously with slight modifications [36]. Briefly, HeLa cells were plated the day before the experiment at a density of 10^6^ cells/100 mm-diameter dish. Cells were challenged with exponentially grown bacteria resuspended in DMEM buffer containing 50 mM HEPES pH 7.4 at a final OD_600_ = 1.0. After incubation for 10 min at 22°C followed by 30 min at 37°C, samples were washed three times in PBS and lysed in ice-cold PBS containing 1 mM AEBSF, 0.1 mM CaCl_2_, 1 mM MgCl_2_, and 0.1% Triton X-100. All subsequent experiments were carried out at 4°C. Samples were scraped, transferred to an Eppendorf tube and subjected to 20 strokes using a dounce homogeneizer. Cell debris and bacteria were removed by two successive centrifugations for 5 min at 13 000×g. Supernatants were subjected to immunoprecipitation using a mix of the anti-IpaC mAb K24, J22 and N9, each at a concentration of 3 µg/ml, or the anti-IpaB antiserum at a 1∶100 dilution, followed by protein-G sepharose. Western-blot analysis were performed using an anti-IpaC antiserum at a 1∶10,000 dilution or the anti-IpaB mAb H16 at a final concentration of 1.5 µg/ml and revealed using the ECL reagent (Amersham Corp.). For the A3F1 T3S reporter, samples were subjected to immunoprecipitation using the anti-IpaA pAb and Western blot analysis using anti-FLAG M2 mAb at 0.2 µg/ml.

### Immunofluorescence analysis

Bacteria grown at exponential phase were treated with poly-L-lysine as described above. HeLa cells were plated onto glass coverslips at a density of 10^5^ cells/24 mm×24 mm coverslip and allowed to spread for 48 h. Cells were challenged with the bacterial suspension at a MOI of 20 bacteria/cell and cells were incubated for 15 min at 37°C. To inhibit F-actin prior to bacterial challenge, cells were incubated in DMEM-HEPES buffer containing cytochalasin D at a final concentration of 1 µg/ml for 30 min at 37°C. Bacteria were allowed to adhere to cells in the same buffer for 10 min at 22°C. Samples were washed three times with PBS to remove unbound bacteria and the actin inhibitor and cells were incubated for the indicated time at 37°C. The Iota two-component toxin was used as a delivery system as previously described, with minor modifications [32]. Briefly, cells were incubated with the Ib component at 30 nM final concentration in EM buffer (120 mM NaCl, 6 mM KCl, 1.2 mM CaCl_2_, 1.6 mM MgCl_2_, 6 mM NaHCO_3_, 9 mM glucose, 25 mM HEPES pH 7.4) for 10 min at 21°C. Ia* or IaC was then added at 30 nM final concentration, samples were incubated for another 10 min at 21°C and shifted at 37°C for the indicated amount of time. Samples were fixed with PBS containing 3.7% paraformaldehyde for 20 minutes, and processed for immunofluorescence staining as described previously [50]. Samples were analyzed using a conventional fluorescence microscope using a 40×objective (BH2-RFCA; Olympus Optical Co., Ltd) or with a confocal laser scanning microscope (Zeiss Axiophot). Actin foci were scored by microscopic observation. The numbers are representative of at least 300 cells scored in 3 independent experiments. For the deconvolution of selected fields, acquisition was performed using a LEICA DMRIBe inverted microscope equipped with a piezzo (Physik Instrumente, GmbH & Co, Karlsruhe, Germany), using a 63×objective (N.A. 1.25). Routinely, stacks of focal planes spaced by 0.2 µm were acquired and processed for PSF-based 3D deconvolution in the Metamorph software 7.0r4 (Universal Imaging) using a fast algorithm. Reconstructions were obtained by applying the average function on the stacks of deconvolved images. For live cells imaging, HeLa cells were transfected using the Fugene reagent (Boehringer-Mannheim) and allowed to express the Src-GFP construct [27] for 16 hours prior to bacterial challenge. Samples were mounted in a microscopic chamber on a LEICA DMRIBe inverted microcoscope in a 37°C-temperature controlled box, connected to a Cool-Snap HQ CCD camera (Roper's Instruments), driven by the Metamorph software 6.1 (Universal Imaging).

## Supporting Information

Figure S1 Effects of the Src inhibitor PP2 on *Shigella* invasion. (A,B) HeLa cells were challenged with bacteria for 15 min at 37°C in the absence or the presence of 10 mM PP2. (A) Samples were processed for immunofluorescence staining of bacterial LPS (red) and F-actin (green). Cells challenged with wild-type Shigella (WT); the non-invasive mxiD mutant (mxiD) in buffer alone or in the presence of 10 mM PP2 (+PP2). Scale bar = 10 mm. (B) Actin foci were scored microscopically. Values correspond to the average of counts obtained for at least 300 cells in three independent experiments. (C) HeLa cells were challenged with wild-type *Shigella* for the indicated time points in the absence or the presence of 10 mM PP2. Cell lysates were prepared and analyzed by anti-phosphotyrosine Western blotting (Materials and Methods). The arrowhead indicates the expected migration of cortactin. PP2 leads to inhibition of *Shigella*-induced actin foci and cortactin phosphorylation.
(9.00 MB TIF)

Figure S2 Src but not Fyn increases *Shigella*-induced actin foci formation in SYF cells. (A–D) Cells were challenged for 15 min at 37°C with bacteria as indicated. SYF: parental SYF cells; SYF/Src: SYF cells transfected with Src-GFP; SYF/Fyn: SYF cells transfected with Fyn-GFP. (A) Samples were fixed and processed for immunofluorescence staining of bacterial LPS (red) and F-actin (cyan). Scale bar = 10 mm. (B) Actin foci were scored microscopically. Values correspond to the average number of actin foci per cell±SEM for at least 300 cells in three independent experiments. (C,D) Representative actin foci induced by wild-type *Shigella* in SYF/Src cells (C) or SYF/Fyn cells (D). GFP fluorescence is shown in green. Scale bar = 2 mm. As opposed to Src-GFP, Fyn-GFP is not recruited at *Shigella* contact sites.
(2.02 MB TIF)

Figure S3 Immunofluorescence detection of IpaC in actin foci induced by wild-type *Shigella*. Stable Src transfectants of HeLa cells were challenged at 37°C for 10 min with the *Shigella* wild-type strain M90T. Samples were fixed and processed for fluorescent staining with rhodamine-phalloidin (actin), anti-IpaC pAb (blue), and anti-Src mAb GD11 (Src). Merge: red, actin; blue, IpaC; green, Src. Reconstructions of deconvolved images from focal planes spaced by 0.2 mm. Scale bar = 2 mm. Src and IpaC localize in an inner area of actin foci.
(0.90 MB TIF)

Figure S4 Src recruitment occurs in *Shigella* mutants defective for the T3S effectors IpaA, IpgB2, and IpgD. HeLa cells were transfected with Src-GFP and challenged at 37°C for 15 min. with the ipaB, ipaA, ipgB2, ipgD, and the SF126 mutant strains. Samples were fixed and processed for anti-LPS immunofluorescence labeling (blue), and F-actin staining (red). Src-GFP fluorescence is shown in green. The images correspond to reconstructions of deconvolved images from focal planes spaced by 0.2 mm. The ipaB mutant is defective at inducing Src recruitment, while all other T3S mutants induce Src recruitment.
(6.21 MB TIF)

Figure S5 The ipaC/pC351 and ipaC/pC1myc *Shigella* strains do not induce Src recruitment. (A) Stable Src transfectants of HeLa cells were challenged at 37°C for 10 min with the *Shigella* wild-type strain M90T (WT), ipaC/pC1 (C1), or ipaC/pC351 strain (C351) at an MOI = 300. Samples were fixed and processed for immunofluorescent staining of Src (green) and IpaC (red). The arrows indicate IpaC and Src co-localization. At the high MOI used, bacteria essentially coat the cell surface (not shown). Even at this high MOI, the C351 construct does not induce Src recruitment. (B) HeLa cells transfected with Src-GFP were challenged for 10 min at 37°C with the ipaC/C1myc strain. Samples were fixed and processed for fluorescent staining with rhodamine-phalloidin (actin) and anti-IpaC (blue) Ab. GFP-fluorescence is shown in green. Panels Src, actin and IpaC: deconvolved images of a single focal plane (Materials and Methods); IpaC+phase: overlay of the IpaC panel with the corresponding focal plane acquired in phase contrast. When quantified, *Shigella* ipaC/pC1myc bacteria did not show significant association with Src-GFP structures (145 bacteria, n = 3), whereas 47.0%±12.5 (SEM) of ipaC/pC1 bacteria showed association with Src-GFP (223 bacteria, n = 3).
(5.96 MB TIF)

Figure S6 The IpaC last 72 carboxyterminal residues containing the C3 insertion at residue 351 (IaC351) fused does not induce actin reorganization. HeLa cells transfected with Src-GFP (green) were challenged with the Iota Ib component and IaC351. Samples were incubated for 15 min at 37°C, fixed, and processed for fluorescent staining of F-actin (red). Scale bar = 10 mm. IaC351 does not induce ruffling or the formation of actin foci (395 cells, n = 3).
(2.62 MB TIF)

Figure S7 Localization of fluorescently labeled Ib in actin-like foci in HeLa cells challenged with IbIaC. HeLa cells transfected with Src-GFP were challenged with the Iota Ib component, fluorescently labeled as previously described [33], and IaC. Samples were processed for fluorescent staining of F-actin. Arrows point at IbIaC-induced actin ruffles. Scale bar = 5 mm. Ib localizes in actin-like foci that are enriched in Src-GFP.
(4.95 MB TIF)

Video S1 Early recruitment of Src-GFP at *Shigella* entry sites. HeLa cells transfected with Src-GFP were challenged with wild-type *Shigella* expressing the AfaE adhesin at 37°C. Dual acquisition of phase contrast and GFP fluorescence images was performed every 15 sec. Movie is shown at 8 frames/sec. Src localizes initially at the intimate bacterial-cell contact site prior to recruitment in bacterial-induced ruffles.
(1.66 MB MOV)

Video S2 Cell contact occurs at one bacterial pole during *Shigella* invasion. Phase-contrast acquisition of HeLa cells challenged with wild-type *Shigella* expressing the AfaE adhesin at 37°C. Images were acquired every 15 sec. Movie is shown at 30 frames/sec.
(0.74 MB MOV)
