# Supplementary figures and images for: The IpaC Carboxyterminal Effector Domain Mediates Src-Dependent Actin Polymerization during Shigella Invasion of Epithelial Cells
Source: PLoS Pathog. 2009 Jan 23;5(1):e1000271. doi: 10.1371/journal.ppat.1000271 (PMC2621354; doi:10.1371/journal.ppat.1000271)

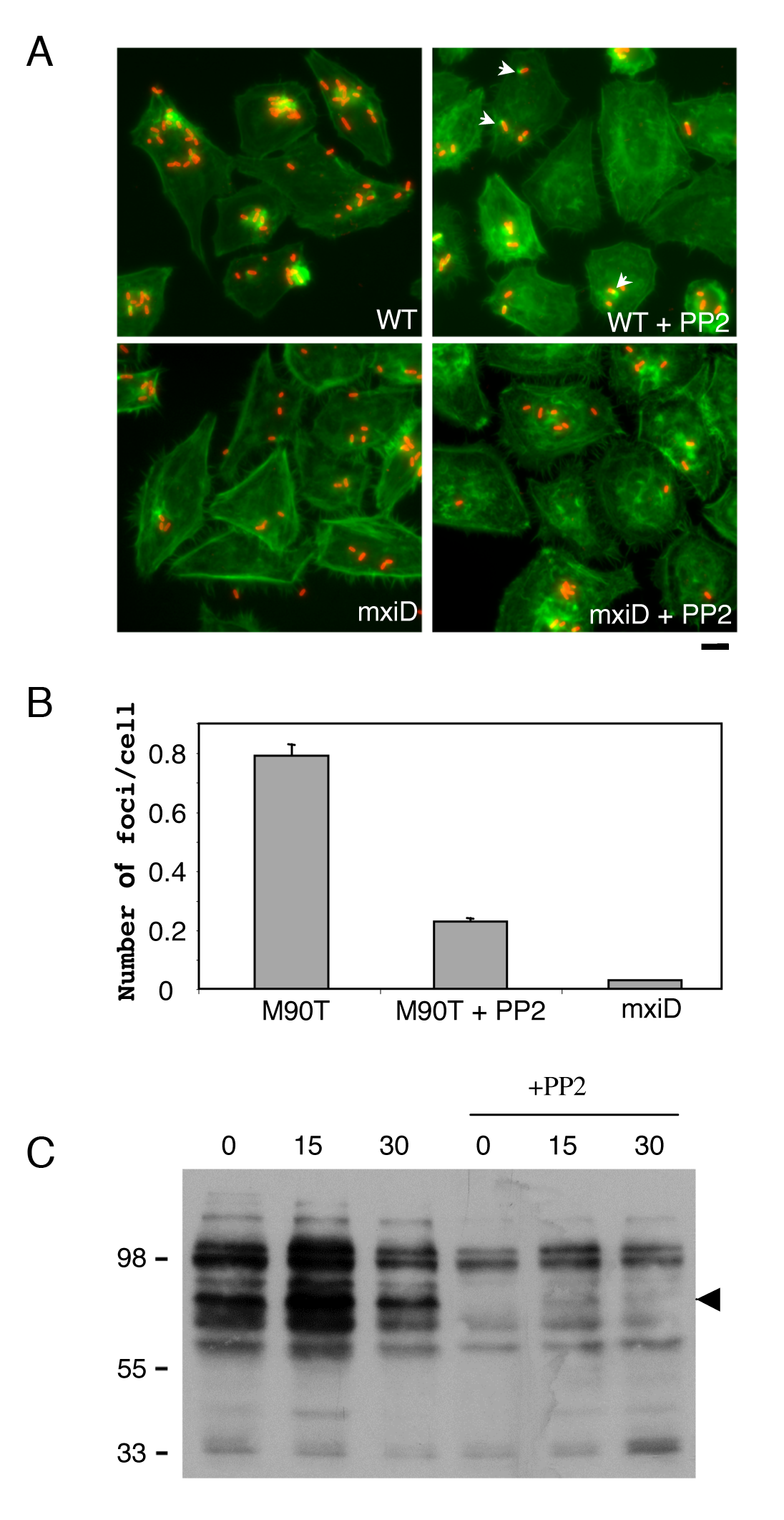

Supplement: Figure S1 — Effects of the Src inhibitor PP2 on Shigella invasion. (A,B) HeLa cells were challenged with bacteria for 15 min at 37°C in the absence or the presence of 10 mM PP2. (A) Samples were processed for immunofluorescence staining of bacterial LPS (red) and F-actin (green). Cells challenged with wild-type Shigella (WT); the non-invasive mxiD mutant (mxiD) in buffer alone or in the presence of 10 mM PP2 (+PP2). Scale bar = 10 mm. (B) Actin foci were scored microscopically. Values correspond to the average of counts obtained for at least 300 cells in three independent experiments. (C) HeLa cells were challenged with wild-type Shigella for the indicated time points in the absence or the presence of 10 mM PP2. Cell lysates were prepared and analyzed by anti-phosphotyrosine Western blotting (Materials and Methods). The arrowhead indicates the expected migration of cortactin. PP2 leads to inhibition of Shigella-induced actin foci and cortactin phosphorylation. (9.00 MB TIF) [file ppat.1000271.s001.tif]

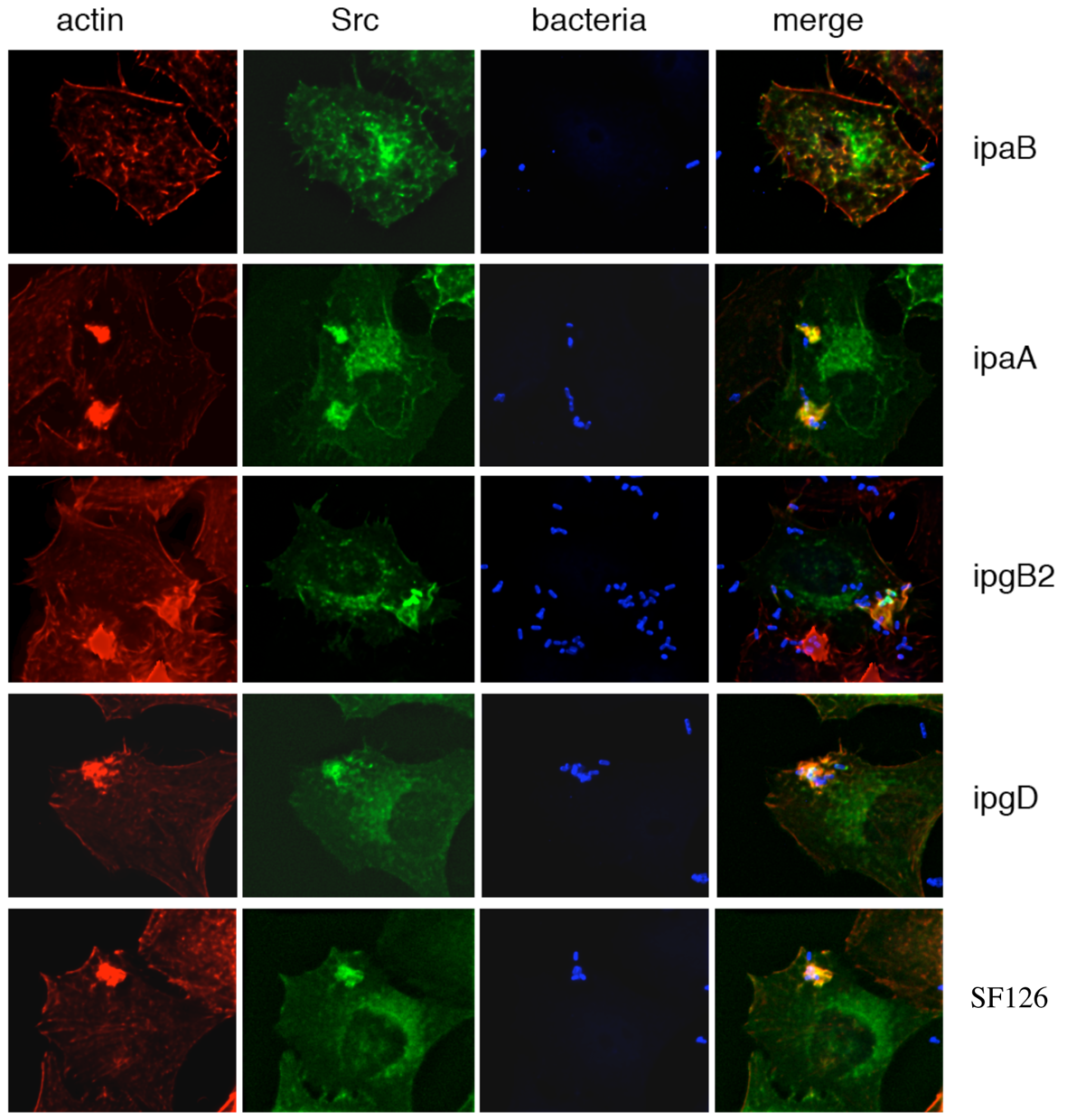

Supplement: Figure S4 — Src recruitment occurs in Shigella mutants defective for the T3S effectors IpaA, IpgB2, and IpgD. HeLa cells were transfected with Src-GFP and challenged at 37°C for 15 min. with the ipaB, ipaA, ipgB2, ipgD, and the SF126 mutant strains. Samples were fixed and processed for anti-LPS immunofluorescence labeling (blue), and F-actin staining (red). Src-GFP fluorescence is shown in green. The images correspond to reconstructions of deconvolved images from focal planes spaced by 0.2 mm. The ipaB mutant is defective at inducing Src recruitment, while all other T3S mutants induce Src recruitment. (6.21 MB TIF) [file ppat.1000271.s004.tif]

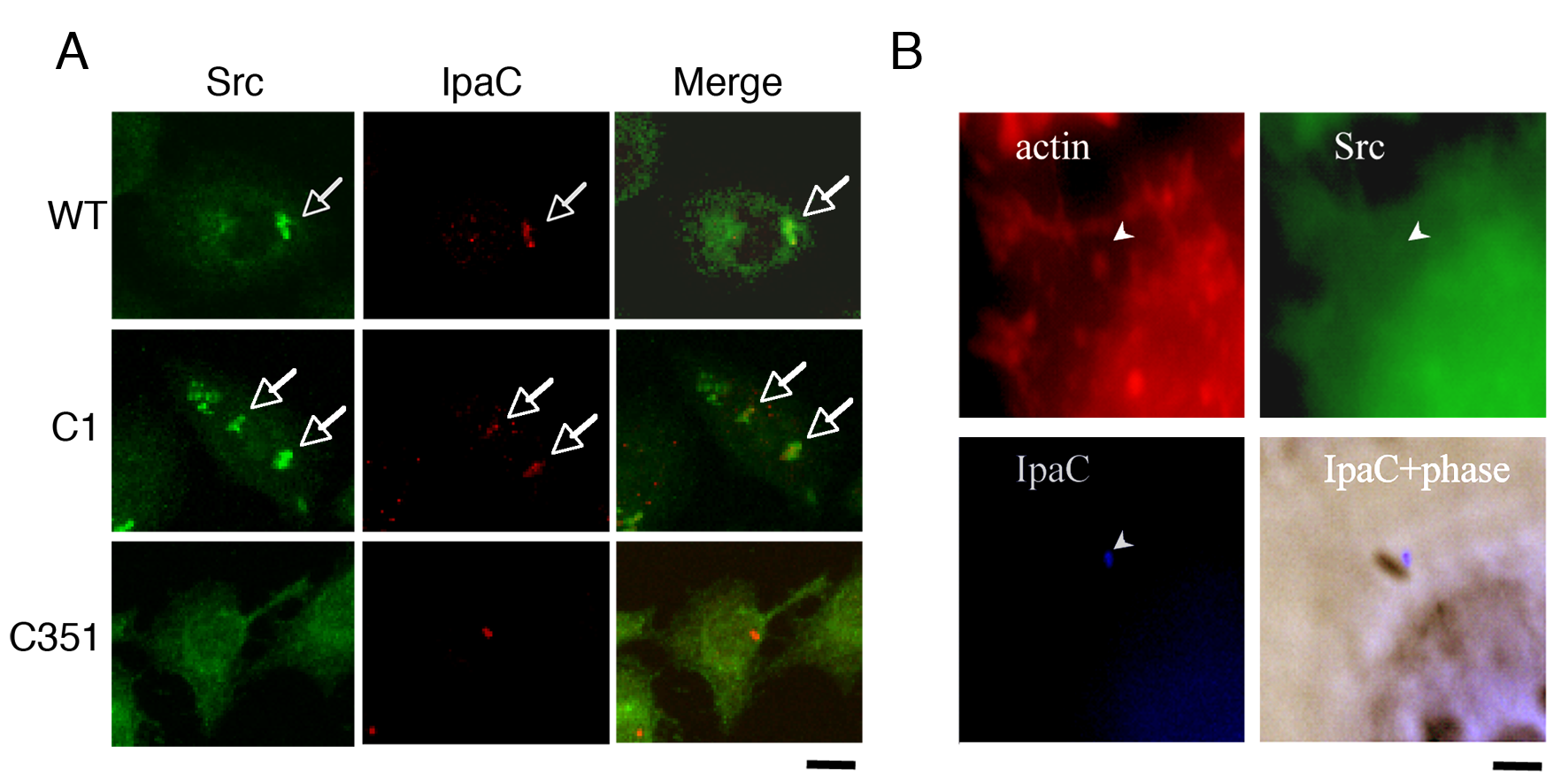

Supplement: Figure S5 — The ipaC/pC351 and ipaC/pC1myc Shigella strains do not induce Src recruitment. (A) Stable Src transfectants of HeLa cells were challenged at 37°C for 10 min with the Shigella wild-type strain M90T (WT), ipaC/pC1 (C1), or ipaC/pC351 strain (C351) at an MOI = 300. Samples were fixed and processed for immunofluorescent staining of Src (green) and IpaC (red). The arrows indicate IpaC and Src co-localization. At the high MOI used, bacteria essentially coat the cell surface (not shown). Even at this high MOI, the C351 construct does not induce Src recruitment. (B) HeLa cells transfected with Src-GFP were challenged for 10 min at 37°C with the ipaC/C1myc strain. Samples were fixed and processed for fluorescent staining with rhodamine-phalloidin (actin) and anti-IpaC (blue) Ab. GFP-fluorescence is shown in green. Panels Src, actin and IpaC: deconvolved images of a single focal plane (Materials and Methods); IpaC+phase: overlay of the IpaC panel with the corresponding focal plane acquired in phase contrast. When quantified, Shigella ipaC/pC1myc bacteria did not show significant association with Src-GFP structures (145 bacteria, n = 3), whereas 47.0%±12.5 (SEM) of ipaC/pC1 bacteria showed association with Src-GFP (223 bacteria, n = 3). (5.96 MB TIF) [file ppat.1000271.s005.tif]

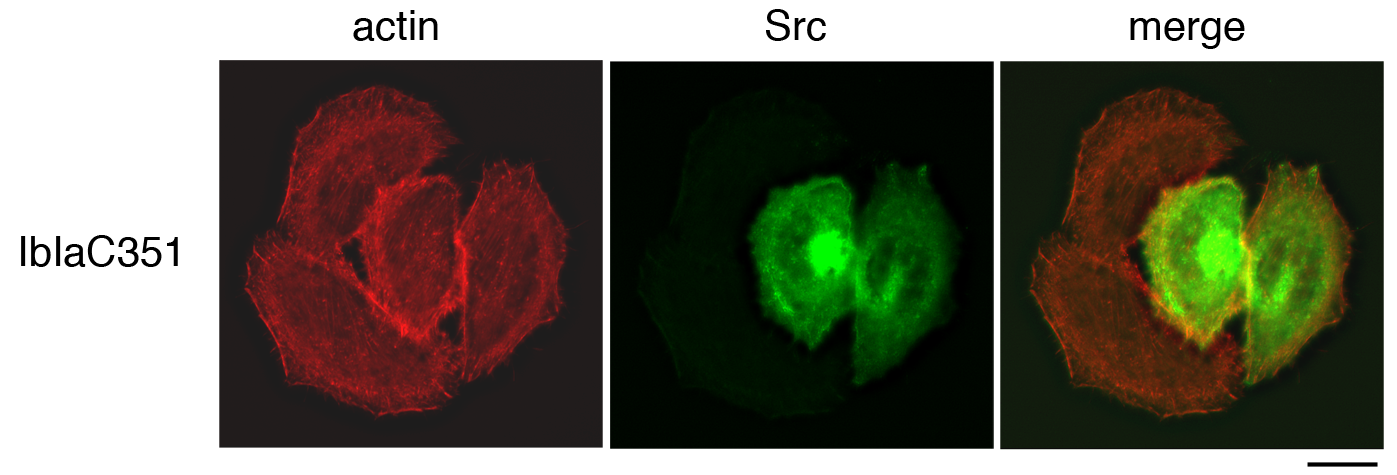

Supplement: Figure S6 — The IpaC last 72 carboxyterminal residues containing the C3 insertion at residue 351 (IaC351) fused does not induce actin reorganization. HeLa cells transfected with Src-GFP (green) were challenged with the Iota Ib component and IaC351. Samples were incubated for 15 min at 37°C, fixed, and processed for fluorescent staining of F-actin (red). Scale bar = 10 mm. IaC351 does not induce ruffling or the formation of actin foci (395 cells, n = 3). (2.62 MB TIF) [file ppat.1000271.s006.tif]
